# Long-Term Regulation of Excitation–Contraction Coupling and Oxidative Stress in Cardiac Myocytes by Pirfenidone

**DOI:** 10.3389/fphys.2018.01801

**Published:** 2018-12-13

**Authors:** Adrián Monsalvo-Villegas, Diana Stephanie Osornio-Garduño, Guillermo Avila

**Affiliations:** Department of Biochemistry, Cinvestav-IPN, Mexico City, Mexico

**Keywords:** intracellular Ca^2+^, Ca^2+^ channel, contractility, CICR, Ca^2+^-induced Ca^2+^ release, cardiomyocyte

## Abstract

Pirfenidone (PFD) is used to treat human pulmonary fibrosis. Its administration to animals with distinct forms of cardiovascular disease results in striking improvement in cardiac performance. Here, its functional impact on cardiac myocytes was investigated. Cells were kept 1–2 days under either control culture conditions or the presence of PFD (1 mM). Subsequently, they were subjected to electrical stimulation to assess the levels of contractility and intracellular Ca^2+^. The PFD treatment promoted an increase in both peak contraction and kinetics of shortening and relaxation. Moreover, the amplitude and kinetics of Ca^2+^ transients were enhanced as well. Excitation–contraction coupling (ECC) was also investigated, under whole-cell patch-clamp conditions. In keeping with a previous report, PFD increased twofold the density of Ca^2+^ current (I_Ca_). Notably, a similar increase in the magnitude of Ca^2+^ transients was also observed. Thus, the gain of ECC was unaltered. Likewise, PFD did not alter the peak amplitude of caffeine-induced Ca^2+^ release, indicating stimulation of Ca^2+^-induced–Ca^2+^-release (CICR) at constant sarcoplasmic reticulum Ca^2+^ load. A phase-plane analysis indicated that PFD promotes myofilament Ca^2+^ desensitization, which is being compensated by higher levels of Ca^2+^ to promote contraction. Interestingly, although the expression of the Na^+^/Ca^2+^ exchanger (NCX) was unaffected, the decay of Ca^2+^ signal in the presence of caffeine was 50% slower in PFD-treated cells (compared with controls), suggesting that PFD downregulates the activity of the exchanger. PFD also inhibited the production of reactive oxygen species, under both, basal conditions and the presence of oxidative insults (acetaldehyde and peroxide hydrogen). Conversely, the production of nitric oxide was either increased (in atrial myocytes) or remained unchanged (in ventricular myocytes). Protein levels of endothelial and neuronal nitric oxide synthases (eNOS and nNOS) were also investigated. eNOS values did not exhibit significant changes. By contrast, a dual regulation was observed for nNOS, which consisted of inhibition and stimulation, in ventricular and atrial myocytes, respectively. In the latter cells, therefore, an up-regulation of nNOS was sufficient to stimulate the synthesis of NO. These findings improve our knowledge of molecular mechanisms of PFD action and may also help in explaining the corresponding cardioprotective effects.

## Introduction

Excitation–contraction coupling (ECC) is of pivotal relevance for cardiac contractility. ECC relies, in turn, on a critical phenomenon known as Ca^2+^-induced–Ca^2+^-release (CICR). An action potential (AP) activates voltage-gated Ca^2+^ channels of the plasma membrane (Ca_V_1.2), allowing entry of extracellular Ca^2+^ (I_Ca_) that activates ryanodine receptors (RyR2) and a consequent release of Ca^2+^ from the sarcoplasmic reticulum (SR). The resulting increase in cytosolic Ca^2+^ (Ca^2+^ transient) activates the contractile machinery, and relaxation occurs as the levels of Ca^2+^ decay thanks to the activity of both the sarco/endoplasmic reticulum Ca^2+^ ATPase (SERCA) and the Na^+^/Ca^2+^ exchanger (NCX; for reviews see Bers, 2002; Eisner et al., 2017; Landstrom et al., 2017).

A small synthetic compound termed pirfenidone (PFD, 185 g/mol) is used to treat human pulmonary fibrosis. Many studies have reported that PFD also improves cardiac performance in animal models of: atrial fibrillation (AF, Lee et al., 2006), myocardial infarction (Nguyen et al., 2010; Li et al., 2017; Adamo et al., 2018), Duchenne muscular dystrophy (Van Erp et al., 2006), pressure overload (Mirkovic et al., 2002; Wang et al., 2013; Yamagami et al., 2015), hypertrophy (Yamazaki et al., 2012), diabetic cardiomyopathy (Miric et al., 2001), and diphtheritic myocarditis (Adamo et al., 2018).

In marked contrast, little is known about the corresponding molecular mechanisms. A reduction in tissue fibrosis and stiffness, attributed to low expression levels of a profibrogenic cytokine (transforming growth factor-beta 1, or TGF-β1) is thought to be involved (Avila et al., 2014; Lopez-de la Mora et al., 2015; Graziani et al., 2018). Nevertheless, some effects do not require lowering levels of TGF-β1 or fibrosis. For example, although PFD improves cardiac contractility in dystrophin-deficient (mdx) mice, this action occurs in the absence of changes in tissue fibrosis or stiffness (Van Erp et al., 2006). In addition, treating cardiac myocytes with PFD (1–2 days) induces a substantial increase in the activity of Ca_V_1.2, by a TGF-β1 independent mechanism (Ramos-Mondragón et al., 2012).

Here, we have investigated whether PFD regulates the function of cardiac myocytes. Our present results show that, indeed, in both atrial and ventricular myocytes, PFD improves the magnitude and kinetics of contractility. This can be explained by a concomitant stimulus on both Ca_V_1.2 and CICR. PFD also elicited a marked decrease in NCX activity and promoted changes in the synthesis rate of cytosolic free radicals [reactive oxygen species (ROS) and nitric oxide (NO)]. Interestingly, the compound also regulated the expression of neuronal NO synthase (nNOS), but not of that corresponding to NCX or eNOS. These findings may be essential to explaining how PFD exerts its cardioprotective effects.

## Materials and Methods

### Isolation of Cardiac Myocytes

Atrial and ventricular myocytes were obtained as described elsewhere (Ramos-Mondragón et al., 2012; Santamaria-Herrera et al., 2016). The procedure was approved by the *Institutional Animal Care and Use Committee* (IACUC—CINVESTAV, 0199-16), complies with the Mexican Official Norm NOM-062-ZOO-1999 and is in accordance with the Guide for the Care and Use of Laboratory Animals published by the US National Institutes of Health (NIH Publication, 8th Edition, 2011).

In brief, male Wistar rats of approximately 240 g were deeply anesthetized, using a mixture of Ketamine and Xylazine (100:10 mg/Kg, i.p.). The heart was then quickly removed by thoracotomy, cannulated (via aorta) and mounted into a homemade Langendorff system. A warm (37°C) digestion buffer containing a mixture of collagenase type II and protease was retrogradely perfused, and the tissue (either ventricles or right atrium) was excised as soon as became softened. Subsequently, samples were carefully triturated, centrifuged and the resulting myocytes were plated into laminin-coated coverslips, which in turn were lying into 35 mm Petri dishes. The cells were transferred to a CO_2_ incubator, kept at 37°C and maintained in the presence of standard culture medium [control (CN)] or culture medium supplemented with PFD (1 mM). Unless otherwise specified, experiments were performed 1–2 days after of have initiated the PFD treatment. This protocol was chosen based on previous determinations of the maximal effect of PFD on Ca_V_1.2 (Ramos-Mondragón et al., 2012).

### Voltage-Clamp Experiments

Ca^2+^ transients and I_Ca_ were investigated simultaneously using a combination of the voltage-clamp technique and epifluorescence (for a more comprehensive description, see Vega et al., 2011). Briefly, either atrial or ventricular myocytes were subjected to voltage-clamp, using the whole-cell patch-clamp technique. The patch-clamp electrodes were filled with an intracellular solution containing a Ca^2+^-sensitive dye (Fluo-4) and exhibited an electrical resistance of approximately 4 MΩ. The cell capacitance (C_m_) was estimated from linear capacitative currents, which were acquired under both cell-attached and whole-cell conditions. Leak currents were eliminated on-line, using a p/N leak subtraction procedure. For each cell, the peak value of I_Ca_ was normalized by C_m_ and plotted as a function of V_m_. The resulting I–V curves were fitted according to the following Boltzmann equation:

(1) $$I_{\text{Ca}}=G_{\text{max}}(V_{\text{m}}-V_{\text{rev}})/\{1+\exp[(V_{1/2}-V_{\text{m}})/k]\}$$

where *G*_max_ represents the maximal Ca^2+^ conductance, *V*_1/2_ is the potential for half-maximal activation of *G*_max_, *k* is a slope factor, and *V*_rev_ is the apparent reversal potential of I_Ca_.

Levels of free cytosolic Ca^2+^ were assessed using Fluo-4. The dye was excited using a 100 W mercury arc lamp (at 470–490 nm), and the emitted fluorescence was collected at 515–550 nm using a photomultiplier tube. The magnitude of Ca^2+^ transients was estimated as the maximum fluorescence value during test pulses (*F*), divided by the basal fluorescence observed before the pulse (*F*_b_), according to: Δ*F*/*F* = (*F* – *F*_b_)/*F*_b_. The gain of EC coupling (ECC Gain) was calculated by normalizing maximal Δ*F*/*F* values to its respective peak value of I_Ca_. The increase in Ca^2+^ signal induced by caffeine (30 mM) was used to estimate the amount of releasable SR Ca^2+^ content. Caffeine was applied via a local, rapid perfusion system (Warner Instruments Corporation), first in the absence and then in the presence of extracellular Na^+^ (see *Recording solutions*). For each cell, the NCX function was examined at the holding potential (−50 mV, atrium; and −40 mV, ventricle) by analyzing Ca^2+^ signals remaining at the end of caffeine applications (25 s).

### Measurements of Contractility and Intracellular Ca^2+^

Ca^2+^ transients and mechanical properties were recorded simultaneously in intact (non-patch clamped) myocytes, with the aid of an IonOptix system (as described elsewhere, Santamaria-Herrera et al., 2016). In short, the cells were loaded with Fura-2 AM (6 μM, 15 min) and then transferred to a stimulation chamber that contained Tyrode’s solution. Subsequently, the chamber was mounted on an inverted microscope equipped with epifluorescence, and electrical stimuli (20 V, 20 ms) were delivery at 0.2 Hz. A particular myocyte was observed on a PC monitor, using a digital video camera. The fluorescence emission ratio of Fura-2 was recorded (F_360_/F_380_), and changes in myocyte length during shortening and re-lengthening were measured at both ends of the cell. In ventricular myocytes, the average sarcomere length was also investigated. Briefly, an area of well-defined striations was selected, and then the average periodicity of Z-line densities was determined (based on a fast Fourier transform algorithm). The sampling frequencies for recordings of contractions and intracellular Ca^2+^ were 0.25 and 1.0 kHz, respectively.

### Intracellular Production of ROS and NO

Production of ROS and NO was estimated using fluorescence sensors for these molecules (CM-H_2_DCF DA and DAF-FM DA; Ríos-Pérez et al., 2016). Either atrial or ventricular myocytes were incubated (15 min at room temperature) with Tyrode’s solution supplemented with a 1:1 mixture of Fura-2 AM and the appropriated sensor (3 μM each). Fura-2 was excited at 360 nm (its isosbestic or Ca^2+^-insensitive point), and its fluorescence was used to normalize signals corresponding to CM-H_2_DCF DA and DAF-FM DA (to prevent for potential differences in loading capacity). Both sensors were excited at 470–490 nm and examined at 515–550 nm.

### Western-Blot Analysis

Protein levels of NCX, nNOS and eNOS, were investigated by western-blot analysis (García-Castañeda et al., 2017). The cells were washed with ice-cold PBS and incubated 15 min in lysis buffer (containing inhibitors of proteases and phosphatases). The total protein concentration was determined by Bradford assay, and protein samples (25–50 μg) were separated by electrophoresis in Laemmli buffer before being electrotransferred to PVDF membranes. The membranes were then blocked with 3% BSA, washed and subjected to immunoblotting with primary antibodies for the protein of interest (at 4°C, overnight). Subsequently, the membranes were incubated with a horseradish peroxidase-conjugated secondary antibody (at 37°C for 60 min). Immunoreactivity was revealed by chemiluminescence, and bands were quantified using Image J (NIH, Bethesda, MD, United States). Each particular membrane was also probed for β-tubulin, to account for potential variability in the amount of protein loaded. The following antibodies were used. *Primary*: anti-NCX (R3F1, Swant, 1:1,000), anti-eNOS (610296, BD Bioscience, 1:800), anti-nNOS (610308, BD Bioscience, 1:800), and anti-β-tubulin (32-2600, Invitrogen, 1:4000). *Secondary*: goat anti-mouse IgG (G-21040, Invitrogen, 1:10,000).

### Recording Solutions

The Tyrode’s solution contained (in mM): NaCl 130, HEPES 25, Glucose 22, KCl 5.4, CaCl_2_ 2, MgCl_2_ 0.5, NaH_2_PO_4_ 0.3, lactic acid 1.1, and pyruvic acid 3 (pH = 7.4).

In atrial myocytes, Ca^2+^ transients and I_Ca_ were recorded in the presence of the following solutions. *External* (in mM): methanesulfonic acid 145, TEA-OH 115, HEPES 10, CaCl_2_ 5, MgSO_4_ 2, 4-aminopyridine 5, 9-anthracene carboxilic acid 1, glucose 10 and 2-3-butanedione monoxime 2. *Internal* (in mM): Cs-Asp 100, CsCl 10, HEPES 10, Cs-EGTA 1, CaCl_2_ 0.2, glucose 10, TEA-HCl 20, ATP-Mg 5, GTP-tris 0.05 and Fluo-4 K_5_ 0.15.

In ventricular myocytes, I_Ca_ and Ca^2+^ transients were recorded in the presence of slightly different solutions (compared with those used for atrial myocytes). *External* (in mM): methanesulfonic acid 125, TEA-OH 115, HEPES 10, CaCl_2_ 10, MgSO_4_ 8, 4-aminopyridine 5, 9-anthracene carboxilic acid 1, 2-aminoethyl diphenylborinate 0.01, Creatine 25, glucose 10 and 2-3-butanedione monoxime 2. *Internal* (in mM): Cs-Asp 160, CsCl 10, HEPES 10, Cs-EGTA 6, CaCl_2_ 1, glucose 10, ATP-Mg 5, TEA-HCl 25, MgCl_2_ 0.5, GTP-tris 0.05 and Fluo-4 K_5_ 0.05.

The NCX activity was investigated by comparing intracellular Ca^2+^ responses triggered by caffeine (30 mM), which was applied using the above-cited *external* solutions (supplemented or not with 140 mM Na^+^. EGTA (0.5 mM) was added as well, to chelate any contaminating Ca^2+^. When required, TEA was omitted to balance the osmolarity.

In all patch-clamp solutions, the pH was adjusted to 7.3.

### Data Analysis

All data were analyzed using the following software packages: SigmaPlot (Systat Software, Inc., San Jose, CA, United States), IonWizard (IonOptix, Westwood, MA, United States), and pCLAMP (Molecular Devices, Sunnyvale, CA, United States). Experimental results are shown as means ± SEM from the indicated number of experiments (n). Two groups were compared by Student’s *t*-test, and ANOVA was used for multiple statistical comparisons.

All experiments were performed at room temperature (∼24°C).

## Results

**FIGURE 1:**
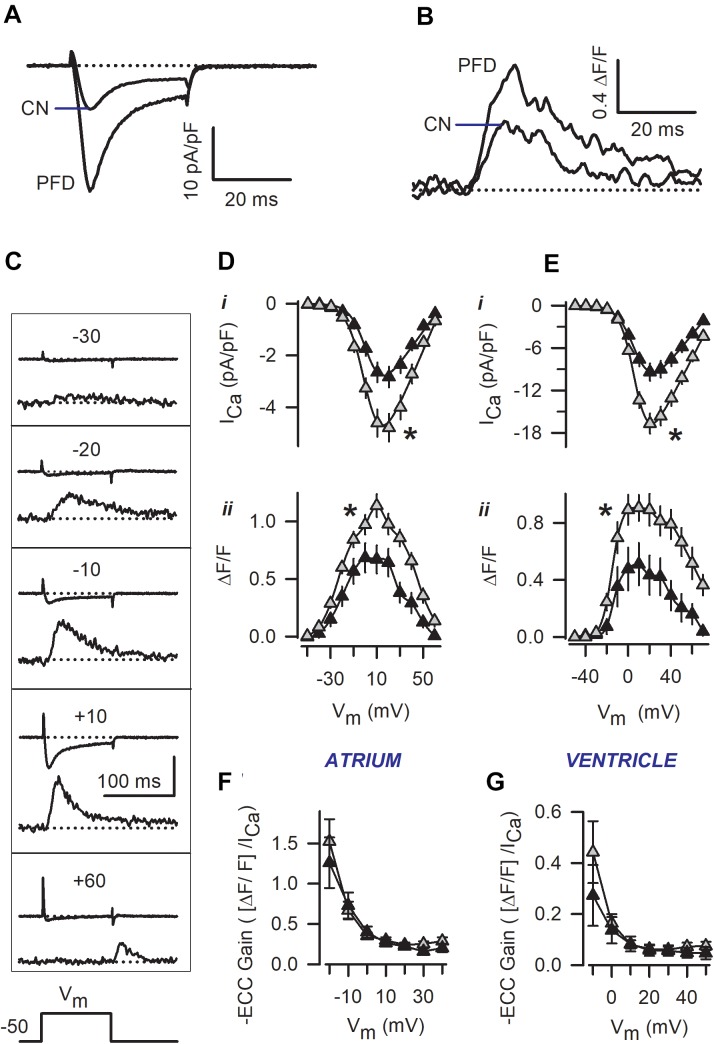
Patch-clamp experiments reveal a pirfenidone (PFD)-induced increase in the magnitude of both I_Ca_ and Ca^2+^ transients. Representative traces of I_Ca_ and Ca^2+^ transients that were recorded simultaneously in either ventricular **(A,B)** or atrial myocytes **(C)**. In **(A,B)**, recordings obtained from control (CN) and PFD-treated (PFD) myocytes were superimposed, and the membrane was depolarized to +10 mV. In contrast, **(C)** illustrates traces obtained at different membrane potentials (V_m_, in a control cell; the vertical calibration bar indicates 100 pA and 1.0 ΔF/F). **(D,E**) Average peak values of I_Ca_ (***i***) and Ca^2+^ transients (***ii***) that were obtained as in **(C)**, for both atrial **(D),** and ventricular **(E)** myocytes. Results are from a total of 28 atrial (11 CN, 17 PFD) and 32 ventricular (17 CN, 15 PFD) myocytes. **(F,G)** Voltage-dependence of ECC gain, estimated from data shown in **(D,E)**. Closed and gray symbols denote control and PFD-treated cells, respectively. ^∗^*p* < 0.05, ANOVA.

Our previous work has shown that the density of I_Ca_ is larger in PFD-treated myocytes than in controls (Ramos-Mondragón et al., 2012). Thus, we first reexamined this aspect, using ventricular myocytes. At the same time, a possible effect on SR Ca^2+^ release was investigated as well. Remarkably, PFD induced a twofold increase in the magnitude of both I_Ca_ and Ca^2+^ transients. More specifically, the following values of I_Ca_ and Ca^2+^ transients were obtained, for control (*n* = 14) and PFD-treated (*n* = 16) myocytes: −9.3 ± 2.0 and −20.9 ± 2.7 pA/pF (*p* < 0.003), and 0.34 ± 0.08 and 0.57 ± 0.06 ΔF/F (*p* < 0.05). Some examples of these determinations are show in Figures 1A,B.

In subsequent experiments, the voltage-dependence of I_Ca_ and Ca^2+^ transients was investigated (see the representative traces of Figure 1C). Interestingly, the PFD effect on both I_Ca_ and Ca^2+^ transients could be observed at different membrane potentials (Figures 1D,E). Fitting I–V curves to a Boltzmann function (Eq. 1) led to the conclusion that PFD causes a 65% increase in the maximal Ca^2+^ conductance (G_max_), in accord with our previous work (Ramos-Mondragón et al., 2012). Specifically, the G_max_ values that were estimated from control and PFD-treated myocytes were: 62 ± 8 and 105 ± 10 nS/pF (*p* < 0.01, *atrium*); and 184 ± 24 and 297 ± 25 nS/pF (*p* < 0.003, *ventricle*). No significant differences were found in the voltage-dependent parameters of G_Ca_ (mid-point and slope factor), between control and PFD-treated myocytes.

**FIGURE 2:**
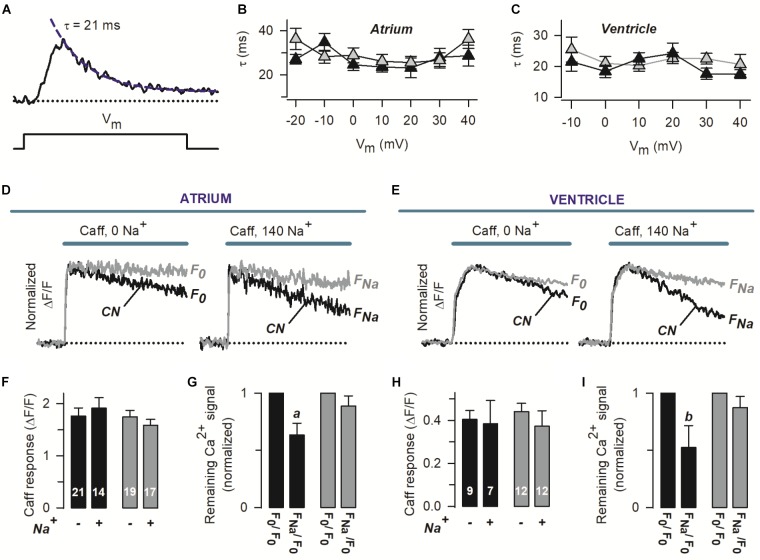
Pirfenidone regulates the rate of Ca^2+^ extrusion via the NCX, but not SERCA activity. **(A)** The panel shows a Ca^2+^ transient recorded as in Figure 1, along with an exponential function (dashed line) describing the decaying phase. The corresponding time constant (τ) is also indicated. **(B,C)** Average values of τ that were obtained as in **(A)**, as a function of V_m_. **(D,E)** Representative Ca^2+^ transients that were induced by a double pulse of caffeine (Caff), initially in the absence (0 Na^+^) and then in the presence of 140 mM extracellular Na^+^ (140 Na^+^). Recordings were obtained from four different myocytes (two atrial–*D* and two ventricular–*E*). F_0_ and F_Na_ stand for the Ca^2+^ signal remaining at the end of caffeine applications, in the absence and presence of Na. The traces are shown normalized to peak caffeine response (F_Peak_), to emphasize kinetic differences. **(F,H)** Absolute average values of F_Peak_ (*Caff response*) and number of investigated cells. **(G,I)** For each particular cell, F_0_ and F_Na_ (see **D,E**) were normalized with respect to F_0_, and the resulting values were pooled and averaged. In all panels, black and gray symbols/traces denote control (CN) and PFD-treated cells, respectively. ^a^*p* < 0.005, *^b^p* < 0.05; compared with the matching F_0_/F_0_ condition (paired *t*-test).

The gain of ECC was also investigated, and it was found identical in control and PFD-treated myocytes (Figures 1F,G). This result suggests that PFD induces an I_Ca_ that is not redundant for ECC. In other words, the extra I_Ca_ elicits the same degree of Ca^2+^ release as its respective control. The activity of SERCA was examined next, as illustrated in Figure 2A. More specifically, the decaying phase of the Ca^2+^ transient was fitted to a simple exponential equation. The resulting average values of τ are shown in Figures 2B,C. It can be appreciated that PDF did not alter this parameter in a wide range of membrane potentials, indicating that PFD does not alter the activity of SERCA.

To assess the amount of releasable SR Ca^2+^, caffeine was applied under both absence and presence of extracellular Na^+^ (see the representative traces shown in Figures 2D,E). Interestingly no significant differences were found in the peak values of caffeine response (*F*_Peak_), between control and PFD-treated myocytes (in either the absence or presence of Na^+^, Figures 2F,H). These data indicate that the effect of PFD on Ca^2+^ transients (Figure 1) is not due to changes in the load of SR Ca^2+^.

Two different concentrations of extracellular Na^+^ were used in Figures 2D–I, because this approach allows investigating both the function of the NCX and Na^+^-independent transporters responsible for Ca^2+^ extrusion. In particular, we examined ΔF/F signals remaining at the end of 25 s of caffeine exposure, in the absence and presence of 140 mM Na (termed *F*_0_ and *F*_Na_, see Figures 2D,E). In the absence of Na^+^, the rate of Ca^2+^ signal decay seems to be slower in PFD-treated cells (Figures 2D,E, *0 Na^+^*). To quantify this observation, for each cell, *F*_0_ was normalized to its corresponding peak caffeine response (*F*_Peak_). The resulting average values were: 0.59 ± 0.04 and 0.78 ± 0.04 (*p* = 0.001), for control (*n* = 30) and PFD-treated cells (*n* = 31). This analysis suggests that PFD inhibits Na^+^-independent Ca^2+^ extrusion systems.

The contribution of Na^+^ to decreasing [Ca^2+^] was next estimated, from *F*_Na_/F_0_ ratios (1.0 means no contribution). Surprisingly, in PFD-treated cells, the NCX activity was not detected (Figures 2G,I, *gray bars*), whereas in control myocytes the exchanger contributed by 45% (Figures 2G,I, *closed bars*). The NCX function was also more directly compared between control and PFD-treated cells (using the *F*_Na_/*F*_0_ values of Figures 2G,I). On average, the *F*_Na_/*F*_0_ values were: 0.60 ± 0.06 and 0.88 ± 0.06 (*p* = 0.012), for control (*n* = 21) and PFD-tread cells (*n* = 29). These data suggest that PFD inhibits the NCX activity by 30%.

**FIGURE 3:**
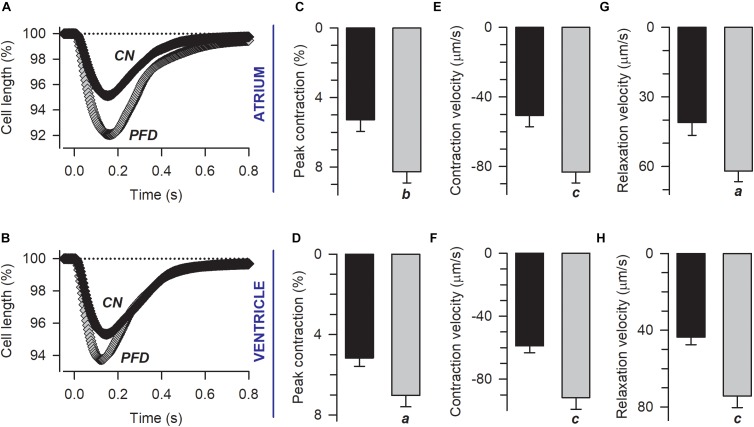
Pirfenidone improves the contractility of cardiac myocytes. **(A,B)** Electrically evoked contractions from atrial **(A)** and ventricular **(B)** myocytes previously kept in culture under either control (CN) or PFD conditions (PFD). Plotted are mean values that were estimated from a total of 56 atrial (30 CN, 26 PFD) and 110 ventricular (59 CN, 51 PFD) myocytes. The corresponding average values (mean ± SEM) of peak cell shortening **(C,D)** and the maximum speed of contraction **(E,F)** and relaxation **(G,H)** are also shown. Black and gray symbols/bars indicate control and PFD-treated cells. ^a^*p* < 0.01, *^b^p* < 0.005, ^c^*p* < 0.001.

Then we wonder if the effects observed in ECC (under non-physiological conditions; i.e., patch-clamp, Figure 1), would have functional consequences. Thus, we investigated the contractility of both sarcomere and cardiomyocytes. Remarkably, PFD increased not only the percent of cell and sarcomere contraction (by 50%) but also the corresponding kinetics of contraction and relaxation (by 30–70%; Figure 3 and Supplementary Figure 1). These PFD effects should lead to improving cardiac blood flow, which is in keeping with results of Nguyen et al. (2010).

**FIGURE 4:**
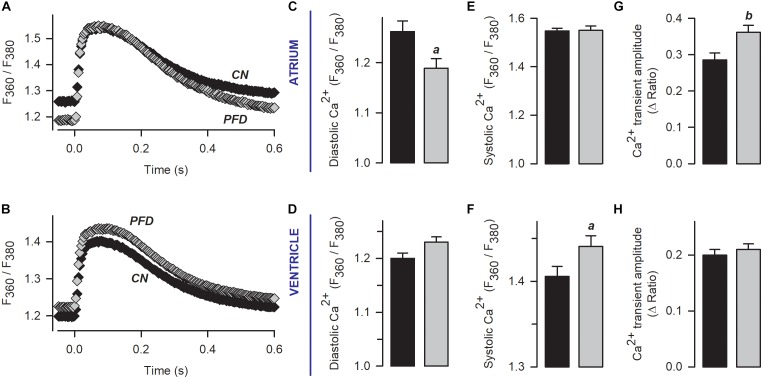
Pirfenidone enhances CICR in intact (non-patch clamped) myocytes. **(A,B)** Mean values of Ca^2+^ transients that were obtained from electrically stimulated atrial (*A*; 42 CN, 37 PFD) and ventricular (*E*; 81 CN, 80 PFD) myocytes. Average values (mean ± SEM) of diastolic **(C,D)** and systolic **(E,F)** Ca^2+^ are also shown, along with the maximum amplitude of the Ca^2+^ transient **(G,H)**. Black and gray symbols/bars indicate control and PFD-treated cells. *^a^p* < 0.05, *^b^p* < 0.01.

In Figure 3, contractility was recorded in parallel with Ca^2+^ transients. An analysis of the corresponding Ca^2+^ signal indicated that, in atrial myocytes, PFD promotes not only Ca^2+^ transients of higher magnitude but also lower values of diastolic Ca^2+^ (compared with controls, Figure 4, *atrium*). With regard to ventricular myocytes, PFD did not alter the amplitude of the Ca^2+^ transient but increased the average values of systolic Ca^2+^ (Figure 4, *ventricle*). Additionally, PFD increased both maximum rate of Ca^2+^ output (in atrium, 21.1 ± 1.6 ΔRatio/s versus 27.5 ± 1.5 ΔRatio/s; *p* < 0.05) and lag time between electrical stimulus and initiation of Ca^2+^ transient decay (in ventricle, 84 ± 4 ms versus 95 ± 4 ms; *p* < 0.05). As a consequence of these changes, the PFD treatment resulted in an increased integral of the initial phase of the Ca^2+^ transient (Supplementary Figure 2). Thus, in intact cardiomyocytes, PFD stimulates CICR and thereby also enhances contractility (Figure 3 and Supplementary Figure 1).

**FIGURE 5:**
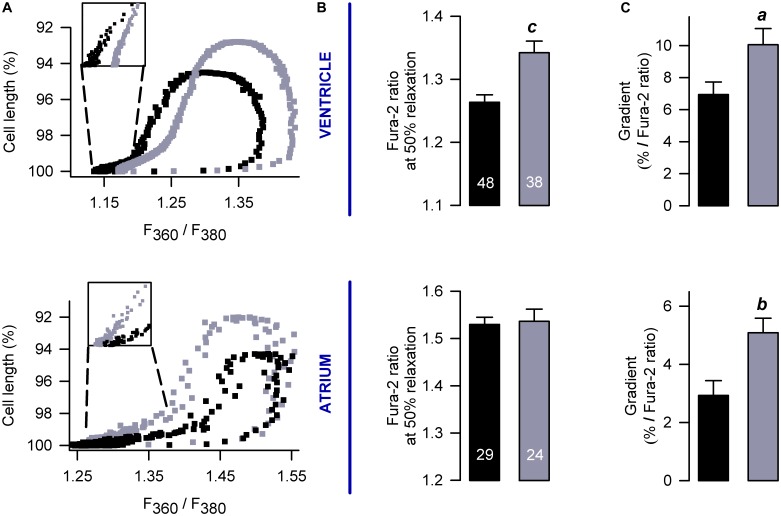
Effects on myofilament Ca^2+^ sensitivity. **(A)** Examples of phase plane diagrams of cell length versus ratio of fluorescence of Fura-2 that were used to analyze myofilament Ca^2+^ sensitivity. Inserts illustrate regions used to estimate the slope of relaxation (or re-lengthening), which were fixed at 90–95% of relaxation. **(B,C)** Average values of Ca^2+^ levels required for 50% of relaxation **(B)** and gradients obtained after fitting endpoint relaxation regions to a straight line (**A**, inserts). The number of investigated cells is shown in **(B)**. ^a^*p* < 0.05, *^b^p* < 0.005, ^c^*p* < 0.001.

We also build phase-plane diagrams, to analyze myofilament Ca^2+^ sensitivity (Figure 5A). The corresponding results indicate that, in the ventricle, PFD not only increases the amplitude of shortening (Figure 3 and Supplementary Figure 1) but also shifts the phase-plane trajectories to the right (Figures 5A,B). Although in atrium a similar shift to the right was absent (Figures 5A,B), an increase in the slope of relaxation was found (Figure 5C). This increase was also present in the ventricle (Figure 5C) and suggests that PFD promotes myofilament Ca^2+^ desensitization (Spurgeon et al., 1992), which is being compensated by higher levels of [Ca^2+^]_i_ to promote contraction (see above).

The cAMP-dependent protein kinase (PKA) phosphorylates key elements of ECC, and thus we investigated whether this protein might be involved in the stimulus of contractility. Ventricular myocytes were exposed 30 min to PKI (a cell-permeable PKA inhibitor), which failed in reverting PFD effects (Supplementary Figure 3). Although in this experiment Ca^2+^ transients were not investigated, the finding that PKI does not interfere with the magnitude or kinetics of cell shortening (Supplementary Figure 3) suggests that myofilament desensitization (Figure 5) is not due to PKA-dependent phosphorylation of cTnI (Cheng and Regnier, 2016).

**FIGURE 6:**
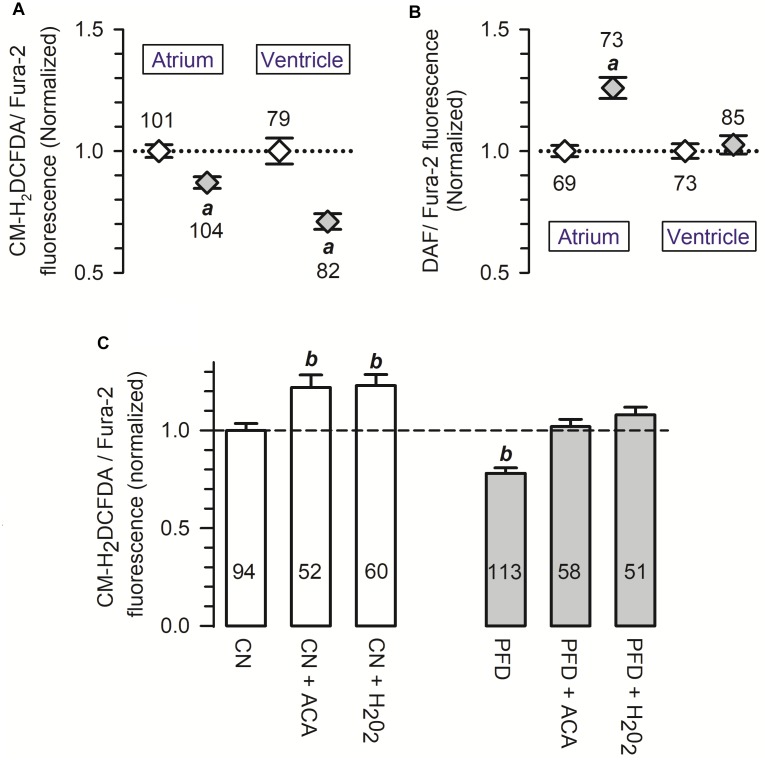
Pirfenidone regulates the production of both ROS and NO. **(A,B)** Cardiomyocytes were loaded with a 1:1 mixture of Fura-2 and a fluorescence sensor for either ROS **(A)** or NO **(B)**. For each cell, the fluorescent signal of the sensor was divided by that of Fura-2 (used as a loading control), and then the resulting ratio was normalized to the respective mean value of control cells. **(C)** Average ROS production measured as in **(A)** (CN, PFD), except that some determinations were performed in the presence of either acetaldehyde (ACA) or H_2_O_2_ (200 μM, 30 min). Open and gray symbols/bars indicate control and PFD-treated treated cells, respectively. ^a^*p* < 0.001, compared with the corresponding control. *^b^p* < 0.005, compared with CN.

Elevated levels of free radicals production are involved in several cardiovascular disorders (for reviews see Zhang et al., 2004; Korantzopoulos et al., 2007; Köhler et al., 2014). Thus, the possibility that PFD regulates the generation of ROS was also investigated. The corresponding results indicate that PFD causes a significant reduction in the signal of a ROS-sensitive fluorescent compound (CM-H_2_DCFDA), revealing a 15–30% inhibition of ROS production (Figure 6A). Conversely, a 1.3-fold upregulation of NO synthesis was also observed, particularly in atrial myocytes (Figure 6B). A minimum of 8 h of PFD-treatment was needed to observe a significant effect on NO and ROS (Supplementary Figure 4), suggesting that changes in protein expression might be involved.

An acute oxidative stress challenge was also investigated, in ventricular myocytes. Specifically, the production of ROS was assessed in cells exposed 30 min to either acetaldehyde (ACA) or hydrogen peroxide (H_2_O_2_). As predicted from results shown in Figure 6A, ROS production was again lower in PFD-treated cells, compared with CN (Figure 6C, *PFD* vs. *CN*). Additionally, in both CN (Figure 6C, *white bars*) and PFD-treated cells (Figure 6C, *gray bars*) the oxidative insults caused a significant increase in ROS production. The production of ROS, however, proved to be identical in the following three experimental conditions: CN, PFD + ACA, and PDF + H_2_O_2_ (Figure 6C). Thus, the results of Figures 6A,C demonstrate that PFD attenuates oxidative stress, not only under basal conditions but also in the presence of pro-oxidants.

**FIGURE 7:**
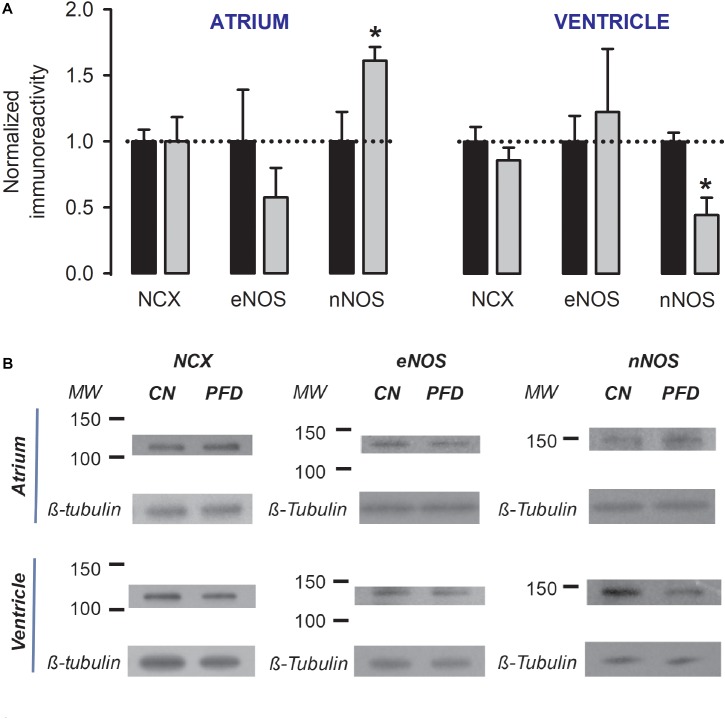
Dual regulation of nNOS expression by PFD. **(A)** Normalized immunoreactivity that was detected in myocytes exposed to primary antibodies against NCX, eNOS, and nNOS (β-tubulin was the loading control). Bars represent the average values from a total of three to four independent experiments. ^∗^*p* < 0.05. **(B)** Examples of western blots that were analyzed to generate data shown in **(A)**.

The regulatory actions on both NCX activity (Figure 2) and NO generation (Figure 6B) can potentially be explained by parallel changes in steady-state expression levels of related proteins. This point was next investigated, by western-blot analysis. PFD did not alter the expression of NCX (Figure 7), suggesting that in PFD-treated cells the disrupted ability of Na^+^ to extrude Ca^2+^ (Figure 2) reflects the lower activity of a fixed number of exchangers.

Pirfenidone also did not alter the expression of eNOS (*p* > 0.3, Figure 7). However, a dual regulation of nNOS was found. The expression level of this enzyme was increased by 60% in atrial myocytes and decreased by 55% in ventricular myocytes (Figure 7). These data suggest that an elevation in expression levels of nNOS (Figure. 7, *atrium*) is sufficient to increase the synthesis of NO (Figure 6B, *atrium*). Conversely, a reduction in these levels (Figure 7, *ventricle*) is not sufficient for changing the production of NO (Figure 6B, *ventricle*).

## Discussion

Cardiac myocytes are of paramount relevance for blood pumping, and its function is compromised in a number of cardiac conditions. On the other hand, many studies show that PFD-treated animals are less prone to develop cardiac dysfunction (Miric et al., 2001; Mirkovic et al., 2002; Lee et al., 2006; Van Erp et al., 2006; Nguyen et al., 2010; Yamazaki et al., 2012; Wang et al., 2013; Yamagami et al., 2015; Li et al., 2017; Adamo et al., 2018). However, no study has systematically investigated the impact of PFD on the function of cardiac myocytes. Consequently, here we have evaluated the influence of PFD on I_Ca_, Ca^2+^ transients, ECC gain, SR Ca^2+^ content, contractility, ROS, NO, and expression levels of some related proteins (NCX, eNOS, and nNOS). Our results demonstrate, for the first time, that PFD upregulates not only Ca_V_1.2 but also CICR and ECC. Remarkably, PFD also: enhances the degree and kinetics of contractility, inhibits Ca^2+^ extrusion via NCX (and other secondary pathways), and lowers oxidative stress.

Below we discuss molecular mechanisms that may underlie our findings, along with the corresponding implications for the *in vivo* condition.

### Molecular Mechanisms of PFD Action

Investigating contractility under physiological-like situations (i.e., in intact myocytes) is essential because the corresponding results are more likely to be significant for *in vivo* conditions. Unfortunately, however, deciphering the underlying mechanisms is complicated because they may comprise several interrelated variables and processes —such as resting membrane potential, the fraction of inactivated channels, the affinity of myofilaments for Ca^2+^, and cytosolic ionic composition (Bers, 2001). On the contrary, interpreting results from voltage-clamp experiments (e.g., under whole-cell patch-clamp) is more straightforward, because many variables either remain constant or are measured (e.g., I_Ca_, C_m_, V_m_, intracellular Ca^2+^, recording solutions). Here we have combined these approaches and obtained evidence suggesting that, by enhancing I_Ca_ and SR Ca^2+^ release, PFD stimulates the contractility of both the sarcomere and cardiomyocytes. We cannot discard, however, a potential contribution of other mechanisms.

Our previous work demonstrated that PDF increases the number of functional Ca_V_1.2 channels (Ramos-Mondragón et al., 2012). This finding, combined with the present observations that the magnitude of Ca^2+^ transients is also enhanced at constant SR load (Figures 1, 2), suggests that PFD prompts the recruitment of Ca^2+^ release units. Atrial and ventricular myocytes are functional and structurally different (Bootman et al., 2011), and thus it will be of interest to investigate the molecular and ultrastructural basis for the recruitment of otherwise “silent” Ca^2+^ release units. This potential mechanism relies on the assumption that, in control cells, Ca^2+^ release units are not saturated by cytosolic Ca^2+^ (even at voltages where I_Ca_ reaches its maximum). Evidence exists indicating that in rat atrial myocytes this happens, even under basal conditions. This is because Ca^2+^ transients elicited by I_Ca_ cannot recruit RyRs located in central regions of the cell, due to the absence of t-tubules. Besides, the mitochondria act as a buffer of Ca^2+^ that complicates free diffusion of the ion, thereby preventing the activation of this “reserve” of RyRs (Mackenzie et al., 2004). The reserve, however, can be recruited under exceptional conditions. For example, during β-adrenergic stimulation and probably also in response to PFD. The following supports this view ***(i)*** PFD inhibits the Ca^2+^ uptake by Na^+^-independent slow systems, which likely include mitochondrial transport; and ***(ii)*** PFD-treated cells must present higher [Ca^2+^]_i_ in microdomains of the cell periphery, due to exacerbated I_Ca_.

A similar model can be proposed for the ventricle. Gómez et al. (1997) reported that hypertrophied ventricular myocytes (derived from hypertensive rats) present a poor ability of I_Ca_ to trigger Ca^2+^ release, in the face of an intact SR Ca^2+^ load. β-adrenergic stimulation reverses this “defective EC coupling,” which likely involves a change of the microarchitecture of the dyad (Gómez et al., 1997). In support of this view, it has been reported that the t-tubular system (and other structures) is altered in hypertrophied hearts (Page and McCallister, 1973). Moreover, biophysical evidence exists supporting the notion that in the hypertrophied myocytes the DHPR may be further from the RyR. More specifically, in these cells, the time course of I_Ca_ inactivation is slowed (Gómez et al., 1997). In our experiments, CN myocytes also likely develop a certain degree of defective EC coupling in culture, which PFD may have prevented. The following supports this hypothesis. Although rat ventricular myocytes retain ultrastructural properties like those of healthy, intact cardiac tissue, for at least during 3 days of culture, subtle structural changes began to appear earlier. For example, within 24 h, intercalated disks became internalized (Mitcheson et al., 1998). Moreover, we found evidence that PFD promotes accelerated kinetics of I_Ca_ inactivation, which represents biophysical evidence of enhanced DHPR/RyR functional interaction (Supplementary Figure 5).

Regarding ventricular myocytes, an apparent inconsistency exists between the results of Figure 1 (whole-cell, patch-clamp) and Figure 4 (intact cells). In particular, PFD increased the magnitude of the Ca^2+^ transient in the former (Figures 1B,E) but not in the latter (Figures 4B,H). Conceivably, different rates of Ca_V_1.2 activation and Ca^2+^ diffusion could have led to obtaining distinct results. These processes are likely more rapid under patch-clamp, because: ***(i)*** in these conditions, membrane depolarization is more expedite than in an AP, and ***(ii)*** the recording solution does not contain Ca^2+^-binding biomolecules (as opposed to the cytosol). Thus, in patch-clamp experiments, PFD probably improved the DHPR/RyR functional interaction more efficiently (see above), because of both enhanced activation kinetics of I_Ca_ and optimized diffusion of the ion. These differences could also account for the observation that in intact ventricular myocytes PFD did not alter the rate of Ca^2+^ output, but instead increased the latency for reaching the peak of the Ca^2+^ transient as well as the corresponding integral (see Figure 4, Supplementary Figure 2 and the corresponding description).

**FIGURE 8:**
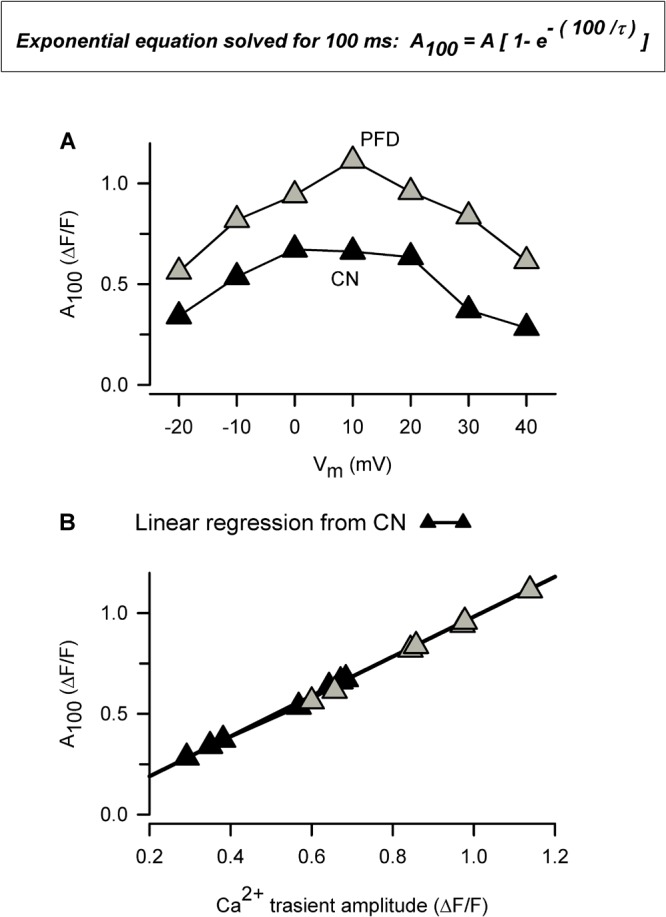
The amount of Ca^2+^ uptake by the SR is higher in PFD-treated cells, compared with controls. **(A)** This plot was built using average values of both τ (Figure 2) and Ca^2+^ transients (Figure 1), according to: A_100_ = A [1- (exp – (t_100_/τ)]; where A is the peak Ca^2+^ transient amplitude, t_100_ represents 100 ms, and A_100_ means the estimated drop in **A** in 100 ms. **(B)** Same data as in a (A_100_) but plotted as a function of peak Ca^2+^ transient amplitude (as opposed to V_m_). Data from exclusively control cells (closed symbols) were fitted according to a linear equation, and the resulting parameters were used to create a theoretical function (solid line; the correlation coefficient, r, was 0.999). Of note, this function describes data from both control and PFD-treated cells (gray symbols), indicating that PFD does not alter SERCA activity but instead feeds the system with the substrate (Ca^2+^).

In response to PFD, the diastolic [Ca^2+^] was decreased in atrial myocytes (Figures 4A,C); however, it showed a tendency to be increased in ventricular myocytes (*p* = 0.08; Figures 4B,D). The reasons for these opposite effects are currently unknown. Additional data combined with a precise mathematical model may eventually help to explain how the fluxes of Ca^2+^ are being balanced. In this respect, our results suggest that PFD-treated cells efficiently compensate for the higher flux of Ca^2+^ into the cytoplasm: ***(i)*** PFD accelerates Ca^2+^-dependent inactivation of Ca_V_1.2. As can be seen in Supplementary Figure 5, PFD promotes a 1.3-fold increase in the percentage of I_Ca_ inactivation. Thus, while the influx of Ca^2+^ via Ca_V_1.2 is higher during the first ∼10 ms, a subsequent accelerated kinetics of inactivation imposes a drastic reduction of Ca^2+^ influx (from at least 30 to 100 ms, Supplementary Figure 5). ***(ii)*** PFD increases the absolute level of SR Ca^2+^ uptake, in proportion to its effects on Ca^2+^ transients. The observation that large and small Ca^2+^ transients return both to basal values with similar time constants (τ) means that the activity of SERCA is unchanged (Figures 2A–C). Nevertheless, it also implies that absolute levels of SR Ca^2+^ uptake are necessarily higher in the former. In other words, our conclusion that PFD does not alter τ (Figures 2A–C) does not mean that SERCA is unable to detect the twofold change in intracellular Ca^2+^ transient amplitude. On the contrary, it can be demonstrated that SERCA detects this change and thereby also accelerates the SR Ca^2+^ uptake. The demonstration consists of estimating the decay of Ca^2+^ transients —in absolute ΔF/F values—, for a fixed period. For example, the decay in ΔF/F per 100 ms is larger in PFD-treated cells than controls (Figure 8A, A_100_). Accordingly, if one displays A_100_ values as a function of the peak Ca^2+^ transient amplitude, it becomes clear that PFD increases the velocity of Ca^2+^ uptake as a consequence of enhancing the magnitude of Ca^2+^ transients (Figure 8B).

Pirfenidone has been the subject of numerous studies regarding long-term *in vivo* effects. Its actions can be grouped as anti-inflammatory, antioxidant and antifibrotic. Changes in expression levels of proteins controlling the development of fibrosis (e.g., TGF-β1), inflammation (e.g., interleukins) and oxidative stress (e.g., catalase) are involved. To date, however, the molecular targets responsible for these changes remain unknown (Avila et al., 2014; Lopez-de la Mora et al., 2015; Graziani et al., 2018). Free radicals are the only PFD-binding molecules identified thus far. In particular, PFD scavenges hydroxyl and superoxide radicals (Giri et al., 1999; Misra and Rabideau, 2000; Mitani et al., 2008). Nevertheless, it is unknown whether this ROS scavenging activity mediates chronic effects.

The present study was designed to characterize long-lasting and persistent modifications of cardiomyocyte function (as opposed to rapid and readily reversible). Hence, cells were exposed to PFD for a minimum of 4 h (at 37°C, in culture medium) and experiments were performed 15–50 min after finishing the treatment (i.e., using extracellular solutions devoid of PFD). Therefore, the changes we have observed persist long after PFD removal. Moreover, at least for the case of ROS and NO production, it took a minimum of 8 h of PFD treatment to detect a significant effect (Supplementary Figure 4).

Thus, most likely in our experiments PFD initially regulated protein expression (e.g., oxidative stress-related proteins and nNOS), and this, in turn, influenced free radicals production and cell function.

Nevertheless, the precise sequence of PFD effects on cardiomyocytes has yet to be firmly stablished, and it is likely that some of them be interrelated. For example, in atrial myocytes, the inhibition of ROS production (Figure 6A and Supplementary Figure 4) could have increased the expression of nNOS (Figure 7), and thereby PFD also induced higher levels of NO synthesis (Figure 6B). The time-course of PFD effects indirectly support these thoughts (Supplementary Figure 4). Specifically, ROS production starts to be regulated after 8 h of PFD-treatment (Supplementary Figure 4B; *atrium*), whereas the stimulation of NO generation requires 24 h to become evident (Supplementary Figure 4D; *atrium*). Moreover, these findings are also in line with results of Kar and collaborators, showing that prolonged exposure of HL-1 atrial myocytes to H_2_O_2_ results in low expression levels of nNOS (Kar et al., 2015). Thus, in atrial myocytes, both the expression of nNOS and NO synthesis seem to be under control of chronic ROS production.

Additionally, it is well-known that ROS stimulates NCX activity (e.g., Goldhaber, 1996; Santacruz-Toloza et al., 2000; Kuster et al., 2010). Remarkably, this effect has been associated with redox modification of the exchanger, which likely interferes with Na^+^-dependent inactivation (Santacruz-Toloza et al., 2000). Thus, in our present control conditions, NCX activity is likely sustained by ROS, and possible removal of redox modification might represent the basis for PFD inhibiting the exchanger. On the other hand, ROS do not appear to be involved in the regulation by PFD of Ca_V_1.2, because in atrial myocytes several classical antioxidants—such as DMSO, glutathione, and *N*-acetylcysteine (NAC)— do not reproduce the PFD effect on I_Ca_ (Ramos-Mondragón et al., 2012). Thus, in addition to ROS (Giri et al., 1999; Misra and Rabideau, 2000; Mitani et al., 2008), PFD also likely acts through other molecular targets (for review see Avila et al., 2014).

In our experiments, PFD inhibited the NCX function, which was estimated 25 s after caffeine stimulation. Thus, it remains unknown whether this effect has an impact on triggered electrical activity. Investigating the NCX by other methods would not only help to solve this question but also to reinforce our conclusions.

We previously speculated that PFD might decrease the production of NO in cardiomyocytes, based (at least partially) on evidence derived from hepatocytes (Nakanishi et al., 2004; Tsuchiya et al., 2004). However, the present study indicates that, in ventricular myocytes, PFD does not alter the NO production rate (despite causing a significant reduction in expression levels of nNOS). Furthermore, in atrial myocytes, the compound increases the production of NO (which is likely associated with the observed increase in nNOS amount). The NO signaling is both relevant and complex (for review, see Simon et al., 2014). This complexity originates, at least partially, in nNOS and eNOS presenting distinct subcellular distribution. Accordingly, it has been proposed that the former primarily modulate RyR and SERCA because of its closer proximity to the SR. Conversely, eNOS is thought to predominantly regulate Ca^2+^ handling proteins near the sarcolemma, such as NCX, sarcolemma Ca^2+^ pump and Ca_V_1.2 (Jian et al., 2014). Additionally, a complex interplay exists between nNOS and ROS. As explained above, nNOS expression and NO levels, both depend on chronic ROS production, whereas nNOS activates, in turn, the ROS-NADPH oxidase 2 (Nox2) signaling (Girouard et al., 2009). Moreover, signaling downstream of NO is divided into two main pathways. One of them involves direct S-nitrosylation (of target proteins), whereas another is characterized by modulation of guanylate cyclase (GC) which in turn regulates cGMP- and cAMP-dependent protein kinases (PKG and PKA). Thus, a systematic study concerning nNOS regulation by PFD (Figure 7A) could begin by using selective modulators of NOS isoforms and protein kinases, combined with measurements of not only global levels of Ca^2+^ and contractility, but also local production/changes of ROS, NO, and Ca^2+^. With regard to PKA, our preliminary results suggest that this kinase might not be involved in up-regulation of contractility (Supplementary Figure 3).

### Potential Pathophysiological Relevance

Regardless of the precise molecular mechanisms involved, the present results are likely to have pathophysiological implications. *In vivo*, PFD decreases the susceptibility to develop arrhythmias (AF and ventricular tachycardia), and these effects are thought to depend on a concomitant inhibition of fibrosis (Lee et al., 2006; Nguyen et al., 2010). Nevertheless, it is possible that functional changes in cardiomyocytes also contribute. The enhanced activity of Ca_V_1.2 could increase the effective refractory period, and thereby decrease propensity toward electrical reentry (Ramos-Mondragón et al., 2012; Avila et al., 2014). Besides, the lower activity of the NCX may be decreasing the likelihood of delayed afterdepolarizations (DADs; Voigt et al., 2012), and hence PFD could also inhibit electrical instability and arrhythmia.

Stimulation of CICR, ECC, and contractility, on the other hand, may be of critical relevance to explaining why PFD improves: (***i)*** ventricular developed pressure in mdx mice (both positive and negative dP/dt; Van Erp et al., 2006) and (***ii)*** left ventricular ejection fraction in rats subjected to myocardial infarction (Nguyen et al., 2010).

The inhibition of ROS production by PFD could be relevant in more general terms, because these free radicals are involved in several cardiac conditions, including arrhythmias, myocardial infarct, hypertrophy, alcoholic cardiomyopathy, and heart failure (for reviews see Zhang et al., 2004; Korantzopoulos et al., 2007; Köhler et al., 2014). Thus, shielding myocytes from oxidant damage is likely essential for PFD in preventing cardiac dysfunction.

## Conclusion

Pirfenidone has been long acknowledged to be of therapeutic potential for a number of heart symptoms. *In vivo*, the underlying mechanisms are likely numerous and interrelated. Here, its effects on isolated myocytes were investigated, limiting influences from additional tissues and cells. Our results show for the first time that PFD stimulates the function of cardiac myocytes. Other *in vivo* effects may be regarded as “auxiliary” because the compound acts on essential components of the cardiac muscle-pump. In particular, it stimulates CICR, ECC, and contractility. Moreover, it also decreases ROS production, suggesting that its effects on myocytes are cytoprotective.

## Author Contributions

DO-G performed experiments and analyzed data. AM-V and GA performed experiments, analyzed data, and wrote the paper.

## Conflict of Interest Statement

The authors declare that the research was conducted in the absence of any commercial or financial relationships that could be construed as a potential conflict of interest.
