# Supplementary material for: Long-Term Regulation of Excitation–Contraction Coupling and Oxidative Stress in Cardiac Myocytes by Pirfenidone
Source: Front Physiol. 2018 Dec 13;9:1801. doi: 10.3389/fphys.2018.01801 (PMC6300477; doi:10.3389/fphys.2018.01801)
Supplement: Supplementary file 1 [file Data_Sheet_1.pdf]

Supplementary Material

Long-term regulation of excitation-contraction coupling and oxidative stress in cardiac myocytes by pirfenidone

Adrián Monsalvo-Villegas, Diana Stephanie Osornio-Garduño, and Guillermo Avila*

*** Correspondence:** Corresponding Author: gavila@cinvestav.mx

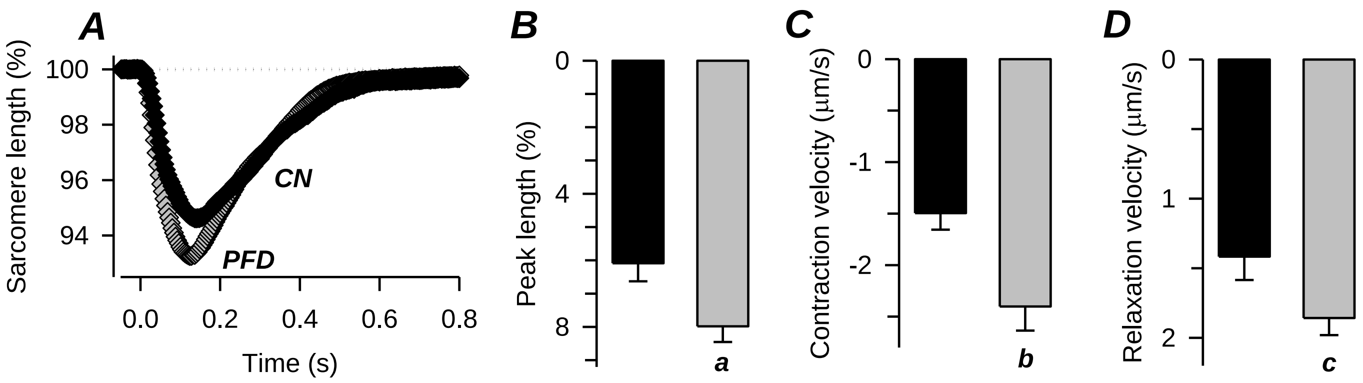

Supplementary Figure 1. PFD stimulates contractile properties of the sarcomere. **A**) Ventricular myocytes were cultured 1-2 d in the absence (CN, *closed symbols*) or presence of PFD (*gray symbols*) and then electrically stimulated to assess the length of the sarcomere as a function of time. Shown are mean values obtained from 129 cells (61 CN, 68 PFD). For each cell, the maximal values of shortening (**B**), and velocities of contraction (**C**) and relaxation (**D**) was estimated as well. [a]p<0.01, [b]p<0.005, [c]p<0.05.

Supplementary Material

Long-term regulation of excitation-contraction coupling and oxidative stress in cardiac myocytes by pirfenidone

Adrián Monsalvo-Villegas, Diana Stephanie Osornio-Garduño, and Guillermo Avila*

*** Correspondence:** Corresponding Author: gavila@cinvestav.mx

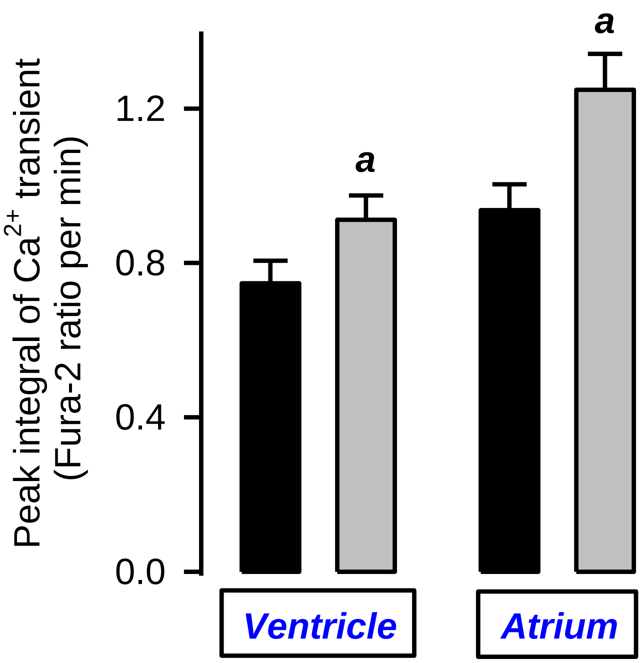

Supplementary Figure 2. Average values of the integral of the initial phase of the Ca^{2+} transient estimated as the area from the stimulus to the peak amplitude. Same cells as in Fig. 4. $^{a}p<0.05$, compared with the corresponding control (closed bars).

Supplementary Material

Long-term regulation of excitation-contraction coupling and oxidative stress in cardiac myocytes by pirfenidone

Adrián Monsalvo-Villegas, Diana Stephanie Osornio-Garduño, and Guillermo Avila*

*** Correspondence:** Corresponding Author: gavila@cinvestav.mx

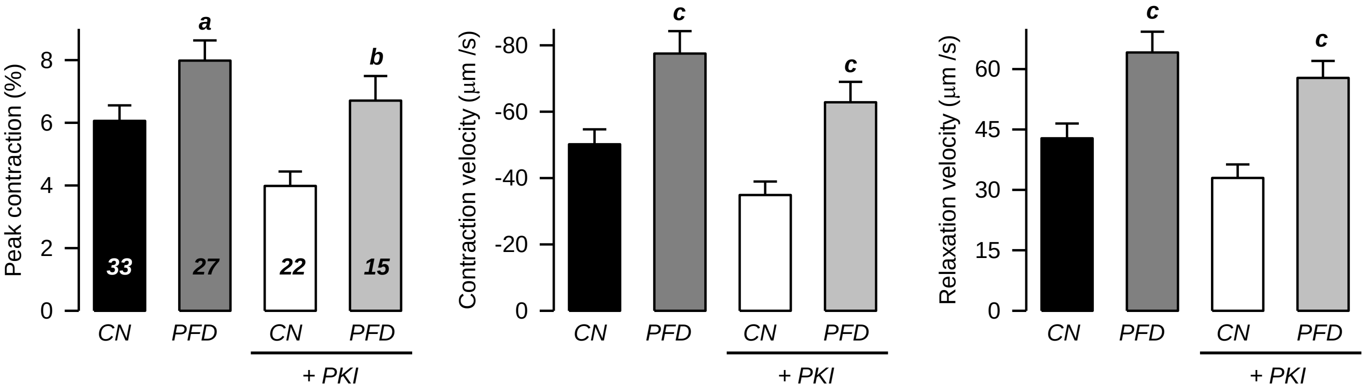

Supplementary Figure 3. PKA is not involved in up-regulation of contractility by PFD. Some control (CN) and PFD-treated ventricular myocytes were exposed 30 min to a cell-permeable PKA inhibitor (myristoylated PKI 14-22 amide, 5 µM). Subsequently, cell contractility was assessed as in Fig. 3. $^{a}p<0.05$, $^{b}p<0.005$, $^{c}p<0.001$; compared with its corresponding control.

Supplementary Material

Long-term regulation of excitation-contraction coupling and oxidative stress in cardiac myocytes by pirfenidone

Adrián Monsalvo-Villegas, Diana Stephanie Osornio-Garduño, and Guillermo Avila*

*** Correspondence:** Corresponding Author: gavila@cinvestav.mx

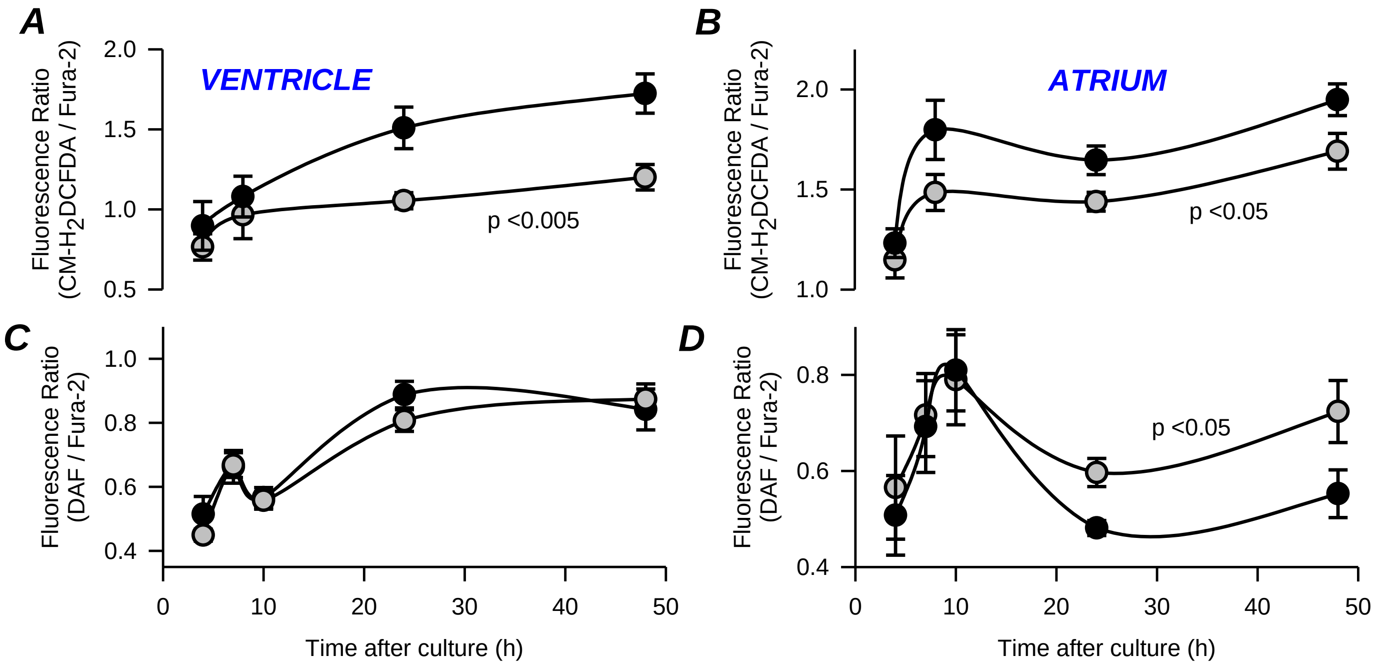

Supplementary Figure 4. Time-course of free radicals production. The panels show fluorescence levels representing the production of ROS (**A, B**) and NO (**C, D**) as a function of time in culture, under either control (*closed symbols*) or PFD (*gray symbols*) conditions. The p values represent significant differences between control and PFD (ANOVA). Each data point was obtained from a total of eight (*D*, 8 h; CN and PFD) to 55 (*A*, 24 h; PFD) myocytes.

Supplementary Material

Long-term regulation of excitation-contraction coupling and oxidative stress in cardiac myocytes by pirfenidone

Adrián Monsalvo-Villegas, Diana Stephanie Osornio-Garduño, and Guillermo Avila*

*** Correspondence:** Corresponding Author: gavila@cinvestav.mx

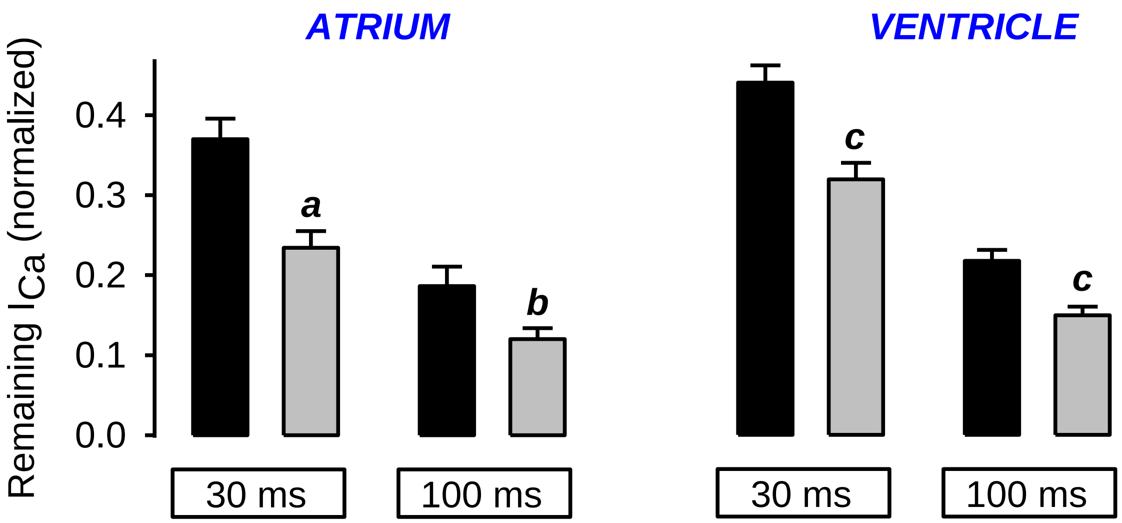

Supplementary Figure 5. PFD promotes accelerated kinetics of I_{Ca} inactivation. Fraction of I_{Ca} that remains not inactivated after 30 and 100 ms of depolarization. Results are from same cells as in Figs. 1D (atrium, at +10 mV) and 1E (ventricle, at +20 mV). a, b and c represent significant differences ($p<0.005$, $p<0.05$ and $p<0.001$), compared with the respective control (closed bars).
